# Synthesis, characterization and evaluation of antimicrobial and cytotoxic activities of biogenic silver nanoparticles synthesized from *Streptomyces xinghaiensis* OF1 strain

**DOI:** 10.1007/s11274-017-2406-3

**Published:** 2018-01-05

**Authors:** Magdalena Wypij, Joanna Czarnecka, Magdalena Świecimska, Hanna Dahm, Mahendra Rai, Patrycja Golinska

**Affiliations:** 1 Department of Microbiology, Nicolaus Copernicus University, Lwowska 1, 87 100 Toruń, Poland; 2 Department of Biochemistry, Nicolaus Copernicus University, Lwowska 1, 87 100 Toruń, Poland; 3 Nanobiotechnology Lab, Department of Biotechnology, SGB Amravati University, Amravati, Maharashtra 444602 India

**Keywords:** Streptomycetes, Biogenic silver nanoparticles, Antimicrobials, Antibacterial activity, Antifungal activity, Synergism, Cytotoxicity

## Abstract

We report synthesis of silver nanoparticles (AgNPs) from *Streptomyces xinghaiensis* OF1 strain, which were characterised by UV–Vis and Fourier transform infrared spectroscopy, Zeta sizer, Nano tracking analyser, and Transmission electron microscopy. The antimicrobial activity of AgNPs alone, and in combination with antibiotics was evaluated against bacteria, namely *Escherichia coli, Pseudomonas aeruginosa, Staphylococcus aureus* and *Bacillus subtilis*, and yeasts viz., *Candida albicans* and *Malassezia furfur* by using micro-dilution method. The minimum inhibitory concentration (MIC) and minimum biocidal concentration of AgNPs against bacterial and yeast strains were determined. Synergistic effect of AgNPs in combination with antibacterial and antifungal antibiotics was determined by FIC index. In addition, MTT assay was performed to study cytotoxicity of AgNPs alone and in combination with antibiotics against mouse fibroblasts and HeLa cell line. Biogenic AgNPs were stable, spherical, small, polydispersed and capped with organic compounds. The variable antimicrobial activity of AgNPs was observed against tested bacteria and yeasts. The lowest MIC (16 µg ml^−1^) of AgNPs was found against *P. aeruginosa*, followed by *C. albicans* and *M. furfur* (both 32 µg ml^−1^), *B. subtilis* and *E. coli* (both 64 µg ml^−1^), and then *S. aureus* and *Klebsiella pneumoniae* (256 µg ml^−1^). The high synergistic effect of antibiotics in combination with AgNPs against tested strains was found. The in vitro cytotoxicity of AgNPs against mouse fibroblasts and cancer HeLa cell lines revealed a dose dependent potential. The IC_50_ value of AgNPs was found in concentrations of 4 and 3.8 µg ml^−1^, respectively. Combination of AgNPs and antibiotics significantly decreased concentrations of both antimicrobials used and retained their high antibacterial and antifungal activity. The synthesis of AgNPs using *S. xinghaiensis* OF1 strain is an eco-friendly, cheap and nontoxic method. The antimicrobial activity of AgNPs could result from their small size. Remarkable synergistic effect of antibiotics and AgNPs offer their valuable potential in nanomedicine for clinical application as a combined therapy in the future.

## Introduction

Due to the extensive application of nanomaterials in medicine, food, agriculture, electronics, energy, etc. their global demand is growing significantly. Silver nanoparticles (AgNPs) are structures at less than 100 nm with unique physical, chemical and biological characteristics. Specific biological activity of AgNPs which results from their physicochemical properties and high surface area to volume ratio have attracted considerable attention globally (Mude et al. 2009).

Nanoparticles are now considered as viable antimicrobial agent and seem to have tremendous potential to solve the problem of microbial multidrug resistance (Rai et al. 2012; Franci et al. 2015) and microbial biofilm formation (Mallevre et al. 2016). Considering limitations of antibiotic therapy and severe side effects of current antimicrobial drugs, there is an urgent need to develop new class of therapeutic agents with better biocompatibility and efficiency.

The biogenic nanoparticles have been found to be active against a wide range of Gram-negative and Gram-positive bacteria such as *Escherichia coli, Vibrio cholerae, Pseudomonas aeruginosa, Salmonella typhi, Listeria monocytogenes, Staphylococcus aureus* and *Bacillus cereus* as well as against fungi including *Candida albicans, Candida tropicalis, Malassezia furfur* and *Trichophyton rubrum* (Morones et al. 2005; Birla et al. 2009; Priyaragini et al. 2013; Anasane et al. 2016; Wypij et al. 2017). The enhanced antimicrobial activity of antibiotics in combination with biogenic AgNPs against *E. coli, P. aeruginosa* and *S. aureus* and fungi such as *C. albicans, C. tropicalis* and dermatophytes causing superficial mycoses, namely *M. furfur* and *T. rubrum* was also observed (Birla et al. 2009; Anasane et al. 2016; Wypij et al. 2017). Therefore, nanoparticles have huge potential for application in medicine (Firdhouse and Lalitha 2015). Moreover, biological synthesis of AgNPs is an easy, efficient and eco-friendly approach as compared to physical or chemical methods (Devi et al. 2012; Rai et al. 2015). In the present study, we selected actinobacterial strain for synthesis of biogenic nanoparticles as these microorganisms are known to be important producers of most natural bioactive compounds, mainly antibiotics and antimetabolites (Bérdy 2005; Newman and Cragg 2007; Olano et al. 2009a, b).

Therefore, the aims of the present study were: (i) synthesize and characterize biogenic AgNPs using actinobacterial strain *Streptomyces xinghaiensis* OF1, (ii) study antimicrobial activity alone and in combination with commonly used antibiotics and antifungal agents, and (iii) evaluate in vitro cytotoxicity against mouse fibroblast and cancer HeLa cell line.

## Materials and methods

### Isolation of OF1 strain from sediment sample

The actinobacterial OF1 strain was isolated from sediment samples of Lonar Crater of Maharashtra, India. The actinobacterium was isolated by serial dilution method as described by Golinska et al. (2013), albeit pH 8.5. Strain was maintained on halophilic nutrient agar (Atlas 2010) slants and as a spore and hyphal fragments in 20% glycerol (v/v) at − 80 °C.

### Molecular identification of OF1 strain

The actinobacterial OF1 strain was identified as *S. xinghaiensis* OF1 strain on the basis of 16S rRNA gene sequence. The search for the closest phylogenetic neighbours based on 16S rRNA gene similarity was performed using the EzTaxon server (http://eztaxon-e.ezbiocloud.net/; Kim et al. 2012). The DNA extraction, PCR amplification and sequencing reactions were performed as previously described by Golinska et al. (2013) and Rathod et al. (2016).

### Synthesis of AgNPs from *S. xinghaiensis* OF1 strain

The actinobacterial *S. xinghaiensis* OF1 strain, was cultured in Erlenmeyer flasks containing 100 ml of halophilic nutrient broth (pH 8.5) and incubated in the orbital shaker (150 r.p.m.) at 27 ± 1 °C for one week. The cell biomass was harvested by centrifugation at 6000×*g* for 10 min and washed thrice with sterile distilled water to remove the attached media components. The cell biomass was re-suspended in 100 ml sterile distilled water, incubated at 27 ± 1 °C for 48 h and then separated by centrifugation 6000×*g* for 10 min. Supernatant was filtered by 0.45 µm cellulose filter, combined with AgNO_3_ solution (final concentration 0.001 mol l^−1^) and incubated at room temperature for 2–3 days. The cell free supernatant without silver nitrate was used as control.

Biosynthesized AgNPs were preliminary detected by colour change of the reaction mixture from colourless to dark-brown, which indicated the formation of AgNPs, and then confirmed by UV–Visible spectroscopy analysis (Nano Drop ND2000, Thermo Scientific, USA) at a resolution of 1 nm by scanning the absorbance spectra in a wavelength range of 200–800 nm. The standard AgNPs (Sigma-Aldrich, size 20 nm and concentration 20 µg ml^−1^) as well as silver nitrate (0.001 mol l^−1^) were also used for UV–Vis spectroscopic analysis.

Solution of biosynthesized AgNPs was treated with NaCl solution (1% v/v) to remove unreacted Ag ions. Biosynthesized nanoparticles present in the solution were centrifuged at 12,000×*g* for 30 min, dried at 40 °C and maintained at 4 °C. The mass of biosynthesized AgNPs was estimated. Proper concentrations of AgNPs were obtained by dissolving AgNPs in sterile, deionized water, medium broth or phosphate-buffered saline (PBS). The AgNPs were active against bacteria and fungi up to 6 months.

### Physico-chemical studies of biogenic AgNPs from *S. xinghaiensis* OF1 strain

The size and topology of biosynthesized AgNPs were characterized by Transmission electron microscopy TEM (FEI Tecnai F20 X-Twintool, USA) after dropping a small volume of AgNP solution on a carbon coated copper grids (400 µm mesh size). The data were calculated by Statistica Software (StatSoft, USA).

The average particle size and size distribution of synthesized AgNPs from *S. xinghaiensis* OF1 strain were analysed by the NTA system LM20 (NanoSight Ltd, UK) after dilution with the nuclease free water. The 0.5 ml of sample was injected onto the sample chamber and observed through LM20.

The stability of biosynthesized AgNPs was estimated based on measurement of their zeta potential using Zeta sizer (Malvern Instruments Ltd, UK). Water was used as a dispersion medium. The refractive index of 1.3, viscosity of 0.8 (cP), dielectric constant of 78.5 and a temperature of − 25 °C were applied and total 12 zeta runs were performed.

The presence of possible biomolecules responsible for the reduction of the Ag^+^ ions and the capping agents over the surface of biosynthesized AgNPs were analysed by Fourier-Transform Infrared Spectroscopy (FTIR; Perkin-Elmer FTIR-2000, USA). The FTIR measurements in the wavelength range of 4000–400 cm^−1^ at a resolution of 4 cm^−1^ were performed after combination of the dry powder of bio-synthesized Ag–nanoparticles with dry KBr (1:100, w/w).

### Minimum inhibitory concentrations (MICs) and minimum biocidal concentrations (MBCs) of AgNPs synthesized from *S. xinghaiensis* OF1 strain against pathogenic bacteria and yeasts

The biosynthesized AgNPs were evaluated against Gram-positive bacteria, namely *S. aureus* (ATCC 6538) and *B. subtilis* (PCM 2021), and Gram-negative bacteria *E. coli* (ATCC 8739), *P. aeruginosa* (ATCC 10145) and *Klebsiella pneumoniae* (ATCC 700603), and against yeasts viz., *C. albicans* (ATCC 10231) and *M. furfur* (DSM 6170).

To determine the MIC of AgNPs, which is defined as the lowest concentration of AgNPs at which there is no visible growth of bacteria or fungi, the broth micro-dilution method recommended by Clinical Laboratory Standards Institute (CLSI) was used (Wayne and Clinical and Laboratory Standards Institute 2012). The assay was performed using 96-well microtiter plates in triplicate. The Tripticase Soy Broth (TSB, Becton Dickinson) and Sabouraud Dextrose Broth (SDB, Becton Dickinson) were used for bacterial and fungal growth, respectively. The SDB supplemented with olive oil (2.5 ml l^−1^) was used for *M. furfur* growth. The final concentration 5 × 10^5^ c.f.u. ml^−1^ of bacteria or fungi was maintained in each well of microtitre plate. The different concentrations (from 0.016 to 1024 µg ml^−1^) of AgNPs were prepared. The positive control (broth mixed with microbial inoculum) and negative control (sterile non-inoculated broth) were also maintained. Microbial inoculum density was estimated by colony counts. Briefly, the microbial inoculum (5 × 10^5^ c.f.u. ml^−1^) was diluted with appropriate broth (1:1000) and 100 µl was then spread on the surface of Tripticase Soy Agar (TSA, Becton Dickinson) or Sabouraud Dextrose Agar (SDA, Becton Dickinson). After incubation, the presence of approximately 50 colonies indicate an inoculum density of 5 × 10^5^ c.f.u. ml^−1^. Inoculated microtitre plates were incubated at 37 °C for 24 h (bacteria and *C. albcans*) or for 48 h (*M. furfur*). The MIC values were then estimated visually.

The minimum biocidal (bactericidal, fungicidal) concentrations (MBCs) of AgNPs against microorganisms, which is defined as the lowest concentration of antimicrobial agent that prevented growth of > 99.9% microbial cells were also determined.

After incubation, 100 µl of each test sample was spread onto antimicrobial agent free medium (TSA for bacteria or SDA for *C. albicans* or SDA with olive oil for *M. furfur*). Plates were incubated at 37 °C for 24 or 48 h and observed for microbial growth.

### Antibiotic susceptibility test for bacteria and yeasts

The susceptibility assay of bacteria to standard antibiotics such as ampicillin, kanamycin and tetracycline; and fungi to amphotericin B, fluconazole and ketoconazole was performed using microdilution method according to CLSI (Wayne and Clinical and Laboratory Standards Institute 2012). The assay was made in triplicate in 96-well microtitre plates as described previously for AgNPs. The antibiotics were tested in concentration range of 0.016–1024 µg ml^−1^. The TSB and SDB were used as diluents for bacteria and fungi, respectively. The final microbial density in each well was 5 × 10^5^ c.f.u. ml^−1^. The MICs of antibiotics were determined visually. The MBCs of antibiotics were determined as described above for AgNPs.

### Biocompatibility index (BI) of AgNPs

BI of AgNPs is defined as ratio of the mean values of IC_50_ of AgNPs on 3T3 and HeLa cells and concentration of AgNPs causing 3 log_10_ reduction in microbial growth (99.9%). A BI higher than 1 indicates that tested compound possess effective microbicidal activity and relatively low cytotoxicity, whereas a BI lower than 1 indicates antimicrobial agent with relatively high cytotoxicity (Müller and Kramer 2008). In the present studies, minimal biocidal concentrations (MBCs) were used for determination of BI as such concentrations caused 99.9% reduction of microbial growth.

### Antimicrobial activity of antibiotics in combination with biosynthesized AgNPs (FIC index determination)

The activity of combination of antimicrobial agents was tested by checkerboard titration method (Saiman 2007) in 96-well microtitre plates (in triplicate). The final concentration of bacteria and fungi in each well was 5 × 10^5^ c.f.u. ml^−1^. The trypticase soy broth (TSB, Becton Dickinson) or Sabouraud dextrose broth (SDB, Becton Dickinson) was used as diluents for bacterial and fungal strains, respectively. The positive and negative controls were maintained. The microtitre plates were incubated at 37 °C for 24 or 48 h (*M. furfur*) to determine the microbial growth inhibition (visually).

Concentrations of antimicrobial and AgNPs used for determination of FIC index were as follows: 2× MICs, 1× MICs, 1/2 MICs, 1/4 MICs, 1/8 MICs, 1/16 MICs.

Based on the data obtained,the fractional inhibitory concentration (FIC) index was determined. The FIC index was calculated by comparing the value of the MIC of each agent alone with the combination-derived MIC. The combination of antimicrobial agents that resulted in at least fourfold reduction in the MIC (1/4 MIC) compared with the MICs of agents alone demonstrated synergistic efficacy (FIC ≤ 0.5). FICs in the > 0.5–1.0 range are considered to be non-synergistic or additive. FICs from 1 to 4 are defined as indifferent, while those of > 4 are antagonistic (Doern 2014).

FIC index formula:

$${\text{FIC}}=\left( {{\text{MIC of AgNPs in combination with antibiotic}}} \right)/({\text{MIC AgNPs alone}})+\left( {{\text{MIC of antibiotic in combination with AgNPs}}} \right)/({\text{MIC of antibiotic alone)}}{\text{.}}$$

### Scanning electron microscopy (SEM) analysis of *E. coli* and *C. albicans* after treatment with AgNPs

SEM (Quanta 3D FEG, Fei, USA) was used for observations of bacterial and fungal cells after treatment with AgNPs. Microbial inoculum (5 × 10^5^ c.f.u. ml^−1^) was combined with AgNP solution (MIC values of AgNPs against *E. coli* and *C. albicans* were used) and incubated for 24 h. The samples were then dropped on a carbon-coated copper grids (400 µm mesh size) and dried at room temperature before analysis.

### Cytotoxic activity of AgNPs

The biogenic AgNPs synthesized from *S. xinghaiensis* OF1 strain were used for in vitro cytotoxicity assay against mouse fibroblasts (3T3) and HeLa cell line.

#### Cell culture

3T3 fibroblasts and HeLa cell line were cultured according to the manufacturer’s protocol under sterile conditions in presence of 4.9% CO_2_ at 37 °C. After thawing, cells were cultured until they reached sub-confluent state. The cells were then detached using 0.25% trypsin solution and seeded into 24-well plates (3 × 10^3^ cells per well) for further experiments.

#### Cytotoxicity evaluation

The cytotoxic activity of AgNPs was evaluated in vitro, using the mouse 3T3 fibroblasts and HeLa cell line in triplicate. The cell viability and proliferative potential based on their metabolic activity were determined with MTT (3-(4,5-dimethylthiazolyl-2)-2,5-diphenyltetrazolium bromide) assay. The adherent culture medium was replaced with medium containing different concentrations (1–100 µg ml^−1^) of AgNPs and incubated for 24 h. Subsequently, the cells were washed with PBS buffer and incubated with MTT reagent (1 mg ml^−1^) at 37 °C for 30 min. The obtained formazan crystals were dissolved in 1 ml of DMSO. The plates were then read spectrophotometrically at a wavelength of 570 nm. The cells were also observed microscopically (Inverted Phase Contrast Microscope Motic AE31 with Moticam and Motic software, Europe).

The percentage growth inhibition was calculated using the formula below:

$$\%\,{\text{Cell inhibition}}=100 - \left\{ {\left( {{\text{At}} - {\text{Ab}}} \right)/\left( {{\text{AcAb}}} \right)} \right\} \times 100$$

where At is the absorbance value of test compound, Ab is absorbance value of blank and Ac is the absorbance value of control.

### Cytotoxic activity of antibiotics

The assay was performed as described above for AgNPs. Antibiotic concentrations used were in the range of 0.016–256 µg ml^−1^. Estimation of percent cell viability was calculated as previously.

### Cytotoxicity of combined antimicrobial agents

Combined antimicrobial agents (AgNPs and proper antibiotic) in concentrations of FIC (given in Table 2), which were found to be active against growth of selected bacterial and fungal strains, were tested for cytotoxicity towards 3T3 and HeLa cells. Cytotoxicity assay was preformed according to details given above for AgNPs. The percent of cell viability was calculated as previously.

**Table 1:** MIC and MBC values (µg ml^−1^) of antibiotics and biogenic AgNPs synthesized from *Streptomyces xinghaiensis* OF1 strain against bacteria and yeasts evaluated by using method of CLSI, and biocompatibility index (BI) of AgNPs

Tested microorganism	MIC of AgNPs	MBC of AgNPs	MIC of antibiotics			MBC of antibiotics			BI of AgNPs	
			AMP	K	TE	AMP	K	TE	3T3	HeLa
*Escherichia coli* ATCC 8739	64	64	> 1024	512	64	> 1024	> 1024	256	0.06	0.06
*Klebsiella pneumoniae* ATCC 700603	256	256	> 1024	192	32	> 1024	512	640	0.016	0.015
*Pseudomonas aeruginosa* ATCC 10145	16	32	384	64	32	> 1024	256	96	0.125	0.12
*Staphylococcus aureus* ATCC 6538	256	384	1	8	4	64	16	256	0.01	0.01
*Bacillus subtilis* PCM 2021	64	64	0.064	2	0.125	0.125	2	48	0.06	0.06

Tested microorganism	MIC of AgNPs	MBC of AgNPs	MIC of antibiotics			MBC of antibiotics			BI of AgNPs	
			AMB	FLU	KCA	AMB	FLU	KCA	3T3	HeLa
*Candida albicans* ATCC 10231	32	> 1024	0.25	> 1024	> 1024	0.5	> 1024	> 1024	0.01	0.01
*Malassezia furfur* DSM 6170	32	48	> 1024	4	> 1024	> 1024	96	> 1024	0.083	0.08

*MIC* minimum inhibitory concentration, *MBC* minimum biocidal concentration, *AMP* ampicillin, *K* kanamycin, *TE* tetracycline, *AMB* amphotericin B, *FLU* fluconazole, *KCA* ketoconazole, *BI* biocompatibility index (IC_50_/rf), *rf* at least 3 log_10_ (99.9%) reduction of microbial growth

## Results

### Molecular identification of actinobacterial OF1 strain

Nearly complete 16S rRNA gene sequence of the isolate OF1 (1414 nucleotides) was determined (GenBank accession number: KY 523106). Isolate was shown to be most closely related to *S. xinghaiensis* S187^T^ sharing a 16S rRNA gene sequence similarity with the closest phylogenetic neighbour of 98.3%, a value that corresponds to 24 nucleotide differences per 1411 locations.

### Biosynthesis of AgNPs from *S. xinghaiensis* OF1 strain

**Fig. 1:**
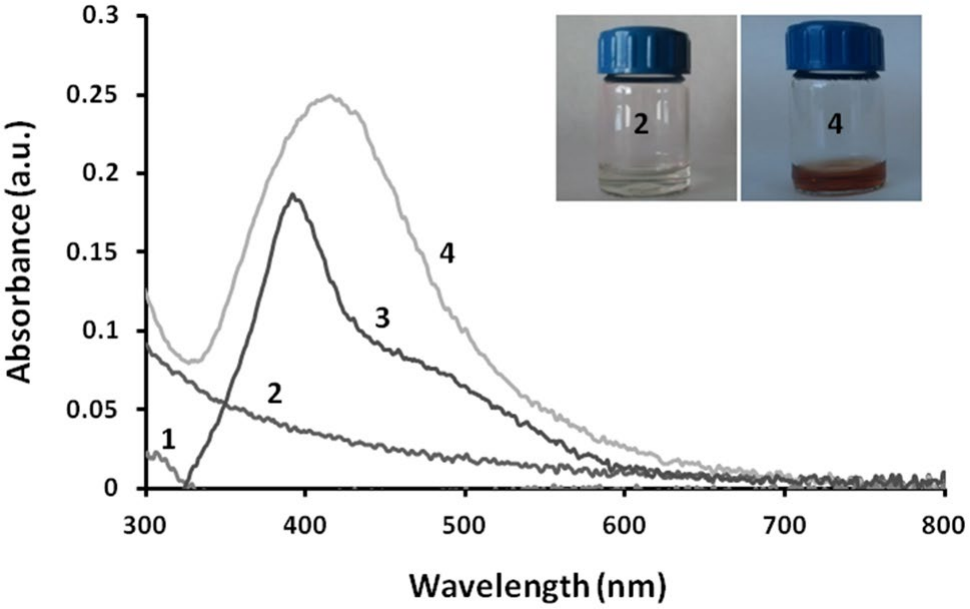
UV–Visible spectrum of silver nanoparticles synthesized from *Streptomyces xinghaiensis* OF1 strain. AgNO_3_ (*1*), control (*2*), standard AgNPs (*3*), and experimental (*4*)

The dark-brown colour of the cell filtrate of strain OF1 after treatment with 1 mM aqueous solution of AgNO_3_ was observed, which confirmed the synthesis of AgNPs. Further, the synthesized AgNPs were characterized by UV–Vis spectroscopy, which revealed narrow peak with a maximum absorbance at 420 nm (Fig. 1).

### Physico-chemical properties of biogenic AgNPs from *S. xinghaiensis* OF1 strain

**Fig. 2:**
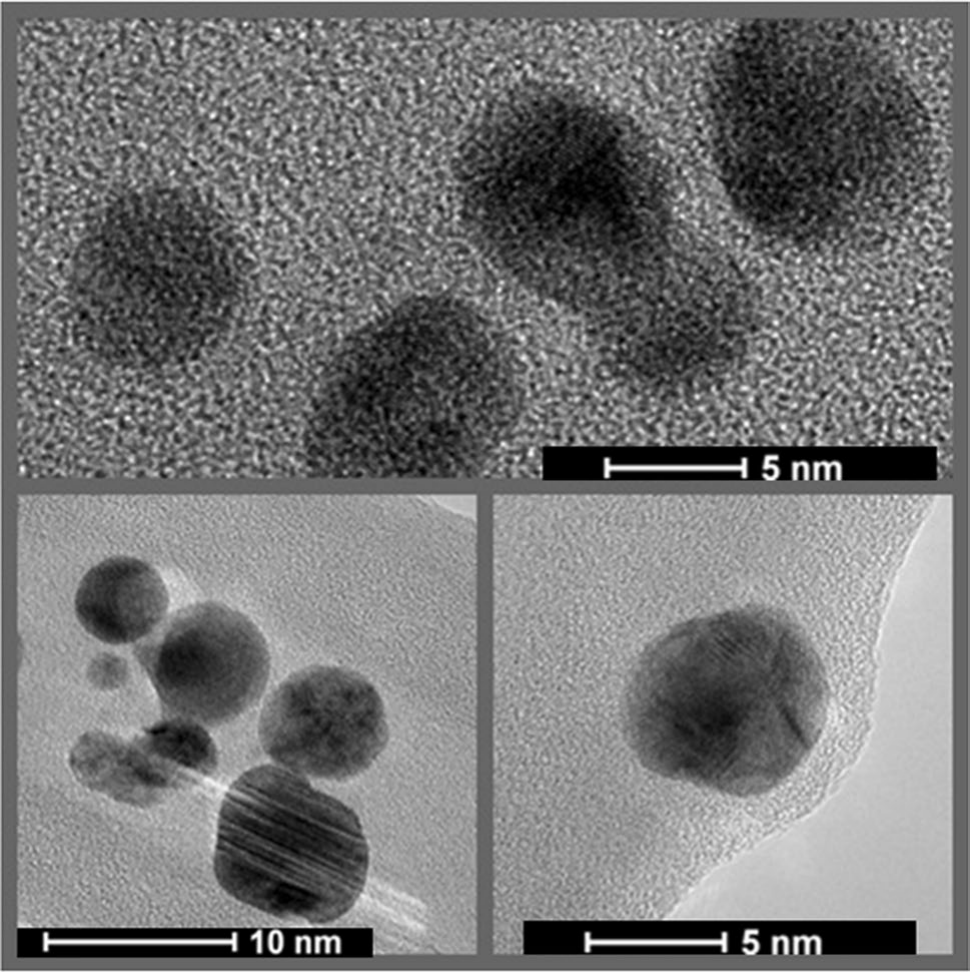
Transmission electron micrograph of silver nanoparticles synthesized from *Streptomyces xinghaiensis* OF1 strain

TEM analysis of AgNPs synthesized from *S. xinghaiensis* OF1 strain revealed the presence of spherical and polydispersed nanoparticles in the size range of 5–20 nm. At some places the aggregation of nanoparticles were also observed (Fig. 2).

**Fig. 3:**
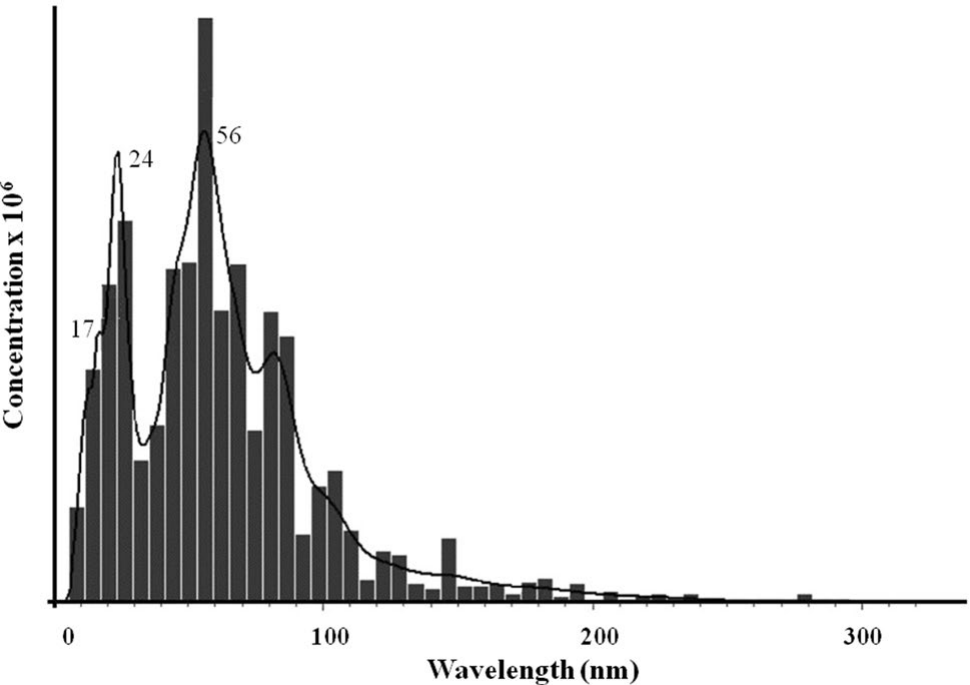
Nanotracking analysis of silver nanoparticles synthesized from *Streptomyces xinghaiensis* OF1 strain

In the present experiment, the nanaoparticle tracking analysis revealed average size of biosynthesized AgNPs of 64 (± 49) nm and their concentration as 2.7 × 10^7^ particles ml^−1^ (Fig. 3).

The zeta potential measurements revealed that the biosynthesized AgNPs were found to be negatively charged (− 15.7 mV).

**Fig. 4:**
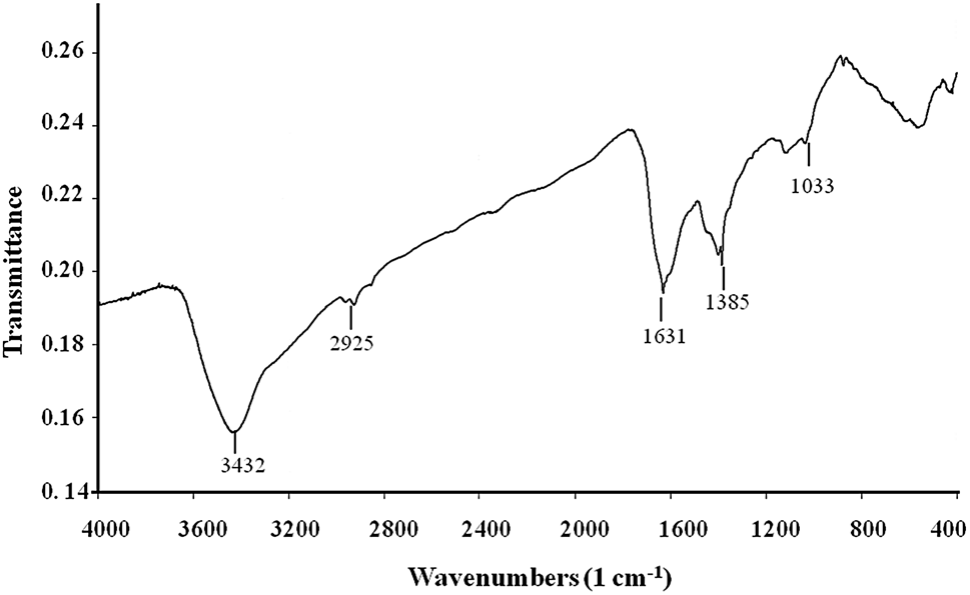
Fourier transform infrared spectroscopy analysis of AgNPs synthesized from *Streptomyces xinghaiensis* OF1 strain. Absorbance bands: 3432, 2925, 1631, 1385 and 1033 cm^−1^

The FTIR analysis of AgNPs synthesized from strain OF1 showed total five absorbance bands at 3432, 2925, 1631, 1385 and 1033 cm^−1^ (Fig. 4).

### Minimum inhibitory concentrations (MICs) and minimum biocidal concentrations (MBCs) of AgNPs from *S. xinghaiensis* OF1 strain against pathogenic bacteria and fungi

The biosynthesized AgNPs from *S. xinghaiensis* OF1 strain exhibited highest inhibitory activity against *P. aeruginosa*, followed by *C. albicans* and *M. furfur*, and then against *B. subtilis* and *E. coli*.* K. pneumoniae* and *S. aureus* were found to be much less sensitive to biosynthesized AgNPs. The MIC values were recorded as follows: 16, 32, 32, 64, 64 and 256 µg ml^−1^, respectively. The minimum biocidal concentration (MBC) of AgNPs was found to be 32 µg ml^−1^ for *P. aeruginosa*, 48 µg ml^−1^ for *M. furfur*, 64 µg ml^−1^ for *E. coli* and *B. subtilis*, 256 µg ml^−1^ for *K. pneumoniae* and 384 µg ml^−1^ for *S. aureus*. MBCs of AgNPs were not recorded for *C. albicans* in tested concentration range (Table 1).

Tested bacteria were most sensitive to tetracycline, followed by kanamycin and ampicillin, while *C. albicans* to amphotericin B, and *M. furfur* to fluconazole.

Biosynthesized AgNPs revealed much higher antibacterial activity than ampicillin (> 1024 µg ml^−1^) against all Gram-negative bacteria and than kanamycin (512 µg ml^−1^) against *E. coli*, and *P. aeruginosa*. However, Gram-positive bacteria, namely *S. aureus* and *B. subtilis* were more susceptible to tested antibiotics in comparison with biosynthesized AgNPs. Similarly, AgNPs from OF1 strain were more active (64 and 16 µg ml^−1^) against *E. coli* and *P. aeruginosa* than kanamycin (> 256 and 64 µg ml^−1^, respectively). Biosynthesized AgNPs were found to be more active against *C. albicans* than fluconazole and ketoconazole (MIC both > 1024 µg ml^−1^) but not than amphotericin B (0.25 µg ml^−1^). Similarly, *M. furfur* was more susceptible to fluconazole than to AgNPs but more resistant to amphotericin B and ketoconazole than to AgNPs (Table 1).

### Efficacy of AgNPs in combination with antimicrobials against bacteria and yeasts

The results of the combination assay are presented in Table 2. Sixteen out of twenty-one antimicrobial agent combinations showed high synergistic effect against tested bacteria and fungi. The FIC index for those combinations was found to be 0.12. Such FIC index value indicates that microbial growth was inhibited in the presence of 1/16 of MIC of both antimicrobials (AgNPs and antimicrobial agent), when compared to antimicrobials used alone. Combination of AgNPs and antibiotics against *P. aeruginosa* revealed indifferent effect on bacterial growth (FIC of 2.0), while combination of amphotericn B and fluconazole with AgNPs against *M. furfur* demonstrated non-synergistic or additive effect (FIC of 1.0) (Table 2).

**Table 2:** Fractional inhibitory concentration (FIC) index determined for AgNPs and antibiotics against bacteria and yeasts

Tested microorganism	FIC index					
	Ampicilin + AgNPs		Kanamycin + AgNPs		Tetracycline + AgNPs	
	FIC	Conc. of antimicrobial and AgNPs used in determined FIC (µg ml^−1^)	FIC	Conc. of antimicrobial and AgNPs used in determined FIC (µg ml^−1^)	FIC	Conc. of antimicrobial and AgNPs used in determined FIC (µg ml^−1^)
*Escherichia coli* ATCC 8739	0.12^a^	64 + **4**	0.12^a^	32 + **4**	0.12	4 + **4**
*Klebsiella pneumoniae* ATCC 700603	0.12^a^	64 + 16	0.12	12 + 16	0.12	2 + 16
*Pseudomonas aeruginosa* ATCC 10145	2.0	768 + 32	2.0	128 + 32	2.0	64 + 32
*Staphylococcus aureus* ATCC 6538	0.12	0.0625 + 16	0.12	0.5 + 16	0.12	0.25 + 16
*Bacillus subtilis* PCM 2021	0.12	0.004 + **4**	0.12	0.125 + **4**	0.12	0.008 + **4**

Tested microorganism	FIC index					
	Amphotericin B + AgNPs		Fluconazole + AgNPs		Ketoconazole + AgNPs	
	FIC	Conc. of antimicrobial and AgNPs used in determined FIC (µg ml^−1^)	FIC	Conc. of antimicrobial and AgNPs used in determined FIC (µg ml^−1^)	FIC	Conc. of antimicrobial and AgNPs used in determined FIC (µg ml^−1^)
*Candida albicans* ATCC 10231	0.12	0.016 + **2**	0.12^a^	64 + **2**	0.12^a^	64 + **2**
*Malassezia furfur* DSM 6170	1.0^a^	1024 + 32	1.0	4 + 32	0.12^a^	64 + **2**

Reduction of AgNPs concentration after combined use with antibiotics to value ≤ IC_50_ (about 4 µg ml^−1^) are highlighted in bold

≤ 0.5 synergism; > 0.5–1.0 non synergistic or additive effect; ≥ 1.0–2.0 indifferent effect (Doern 2014)

^a^As the MIC was not determined in the wide concentration range of antibiotic (see Table 1), the highest concentration of antibiotic was used for FIC index determination

**Fig. 5:**
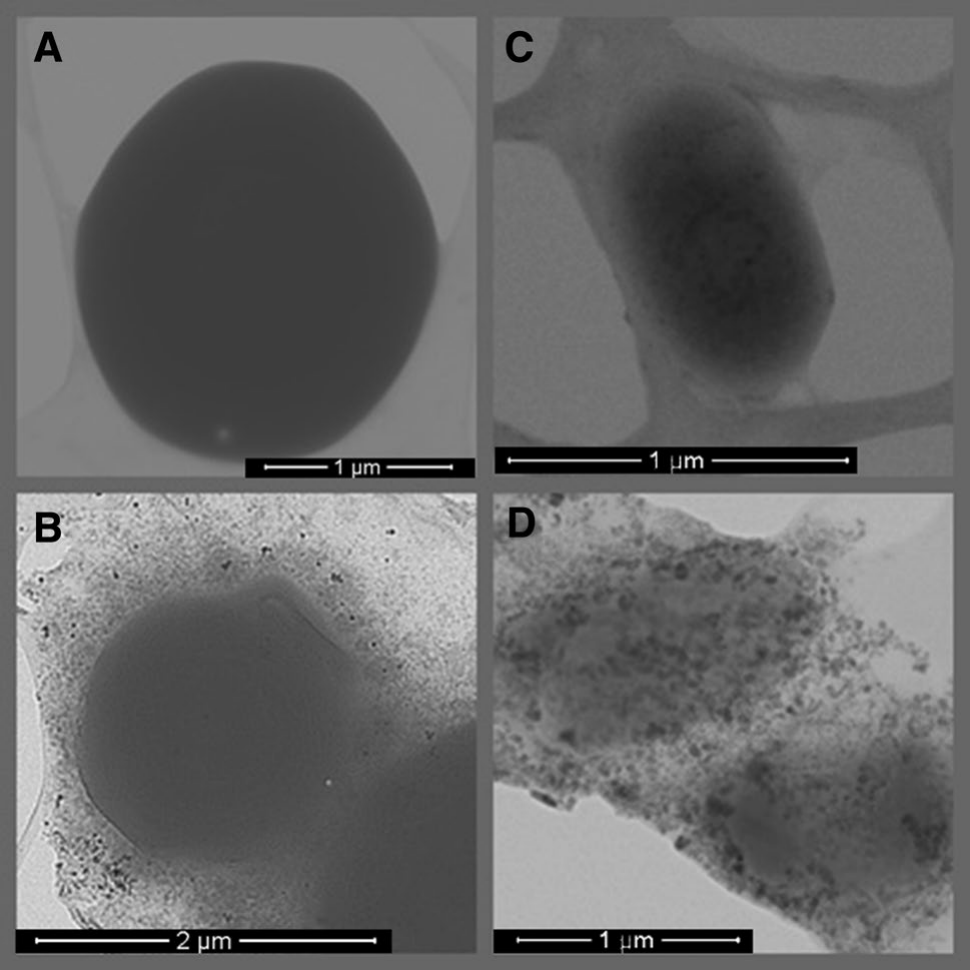
SEM analysis of *Candida albicans* and *Escherichia coli* cells before and after treatment with silver nanoparticles. Upper panel is control and lower panel is microbial cells treated with AgNPs. *C. albicans* (**a, b**) and *E. coli* (**c, d**)

SEM analysis of *E. coli* and *C. albicans* cells after treatment with biosynthesized AgNPs are presented in Fig. 5. The high accumulation of AgNPs around surface of treated bacterial and fungal cells was observed after 24 h of incubation.

**Fig. 6:**
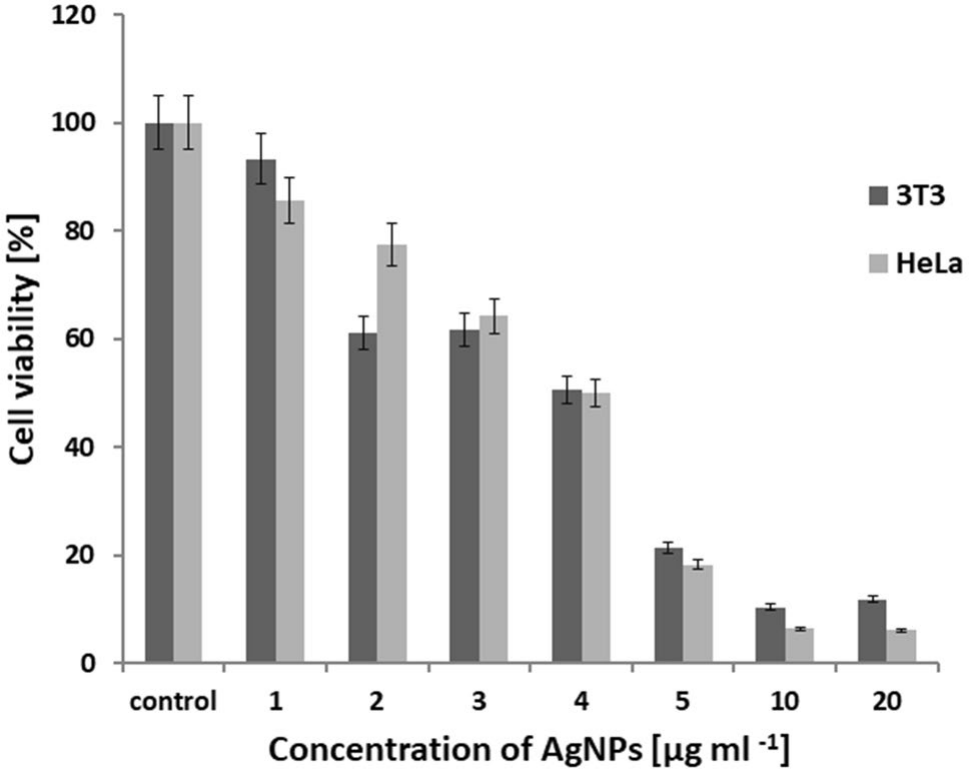
Cytotoxic activity of AgNPs from *Streptomyces xinghaiensis* OF1 strain against mouse fibroblasts (3T3) and HeLa cell line

**Fig. 7:**
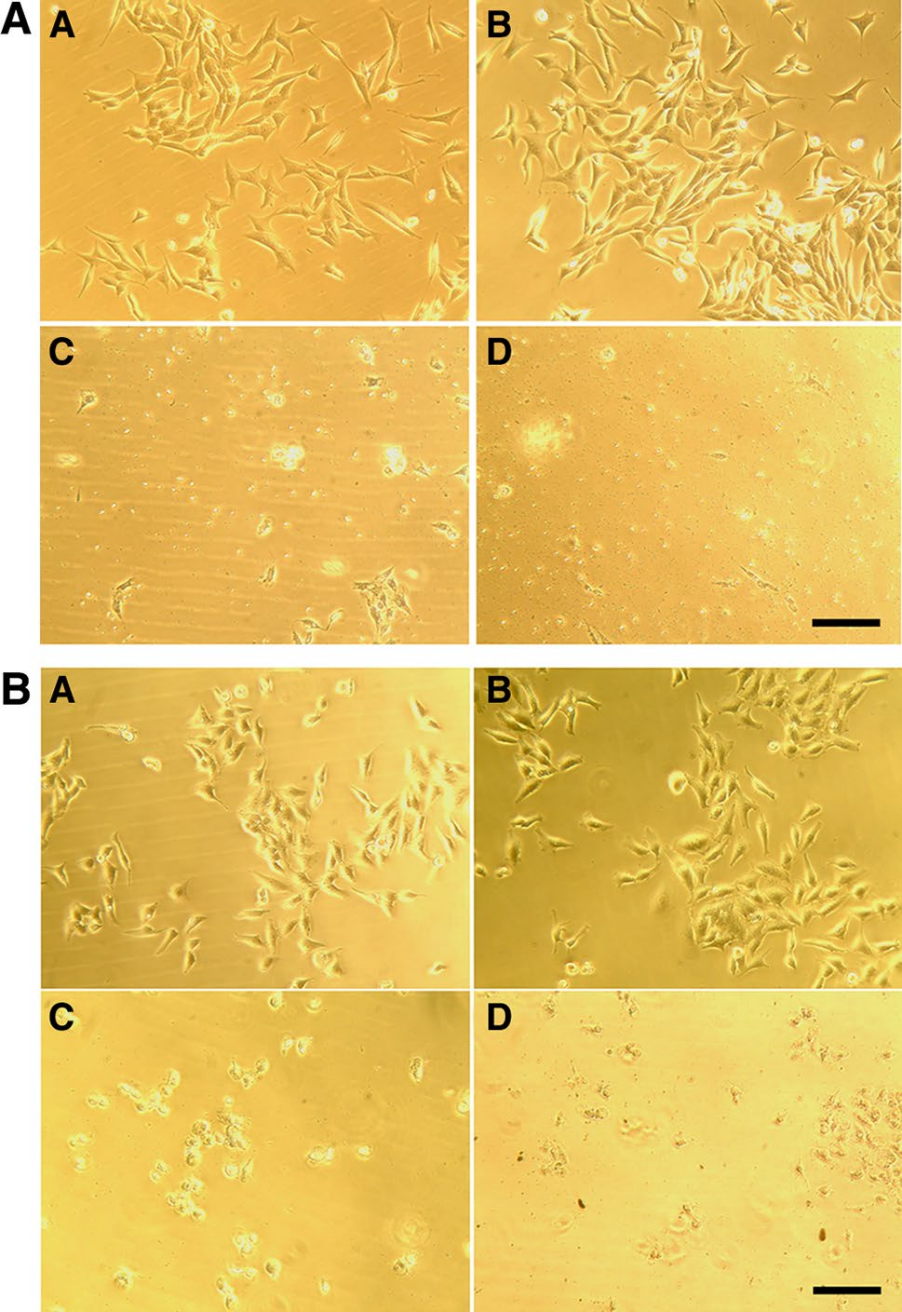
Microscopic observations of mouse fibroblasts (**A**) and HeLa cells (**B**) after treatment with various concentrations of biosynthesized silver nanoparticles (*a* 1 µg ml^−1^, *b* 5 µg ml^−1^, *c* 10 µg ml^−1^, *d* 20 µg ml^−1^). Scale bar 100 µm

The biosynthesized AgNPs from *S. xinghaiensis* OF1 strain were subjected to cytotoxicity on mouse fibroblasts (3T3) and HeLa cell line. The dose dependent effect of AgNPs against eukaryotic cells was observed. The mouse fibroblasts viability after treatment with 1–5, 10 and 20 µg ml^−1^ of AgNPs was found to be 85.5, 61.1, 61.6, 50.5, 18.3, 6.5 and 6.2%, while HeLa cells 93.2, 77.4, 64.3, 50.0, 21.4, 10.45 and 11.9%, respectively (Figs. 6, 7). The IC_50_ value of AgNPs was found to be 4 and 3.8 µg ml^−1^, respectively. The relatively high cytotoxicity of AgNPs was confirmed by biocompatibility index (BI) determination as its values were found to be much lower than 1 (Table 2).

Generally, no toxicity or low toxicity of antibiotics was noticed against mouse fibroblasts (3T3) and HeLa cell line in test concentration range (0.016–256 µg ml^−1)^. There was no toxic effect of kanamycin, amphotericin B, fluconazole and ketoconazole on HeLa cells in tested concentration range, while IC_50_ of ampicillin and tetracycline was found to be at concentrations of 196 and 256 µg ml^−1^, respectively. Similarly, nontoxic effect of kanamycin, tetracycline, amphotericin B and ketoconazole was observed on 3T3 cells, whereas IC_50_ of ampicillin and fluconazole at concentration of 128 and 196 µg ml^−1^, respectively.

**Fig. 8:**
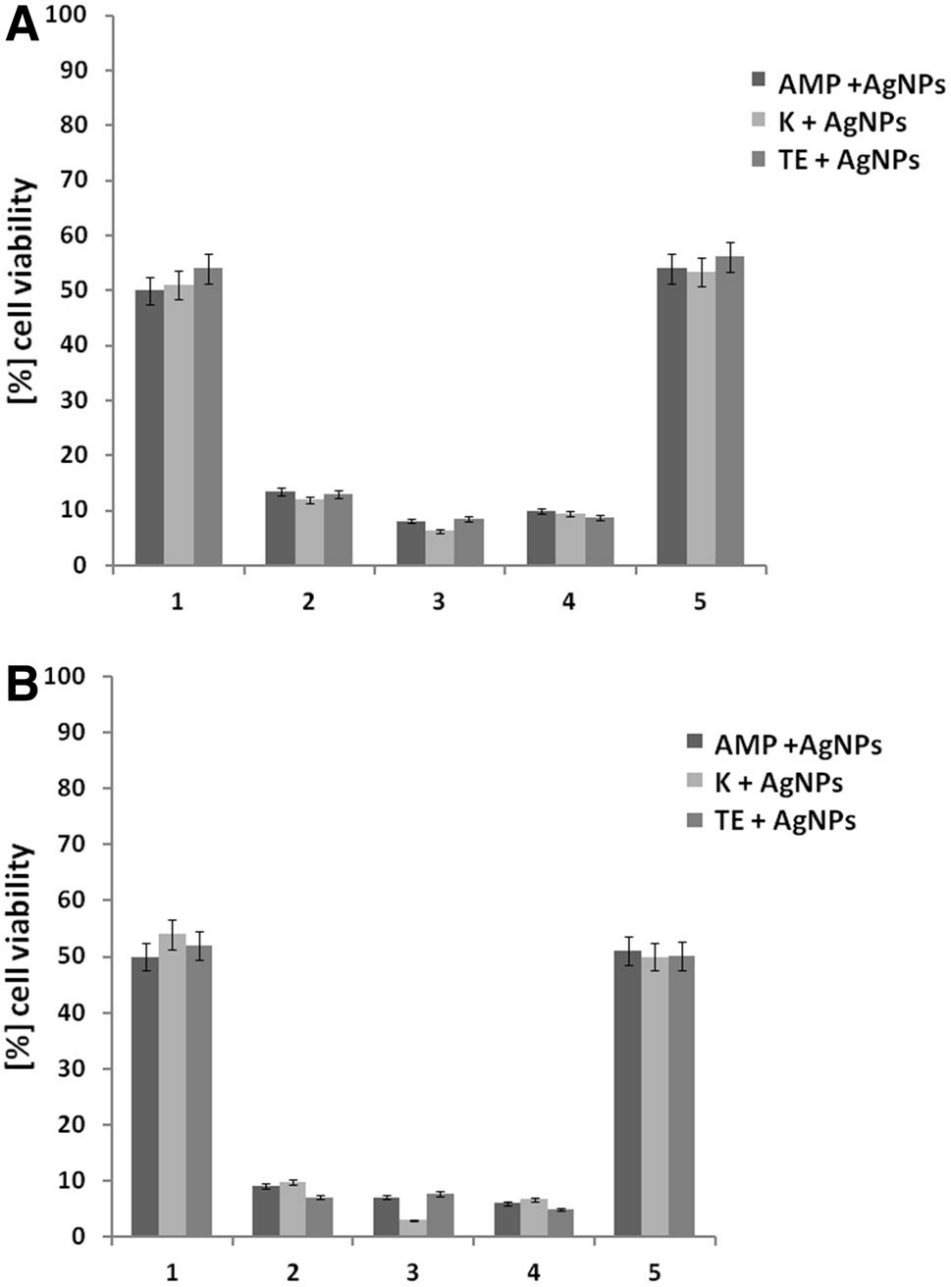
Cytotoxic activity of combined antimicrobial agents (AgNPs and antibacterial antibiotics) against **a** mouse fibroblasts (3T3) and **b** HeLa cell line. Both antimicrobials were used at concentrations of their FIC (see Table 2): (*1*) (4 µg ml^−1^ of AgNPs + 64 µg ml^−1^ AMP or 32 µg ml^−1^ K or 4 µg ml^−1^ TE), (*2*) (16 µg ml^−1^ of AgNPs + 64 µg ml^−1^ AMP or 12 µg ml^−1^ K or 2 µg ml^−1^ TE), (*3*) (1 µg ml^−1^ of AgNPs + 768 µg ml^−1^ AMP or 128 µg ml^−1^ K or 64 µg ml^−1^ TE), (*4*) (16 µg ml^−1^ of AgNPs + 0.06 µg ml^−1^ AMP or 0.5 µg ml^−1^ K or 0.25 µg ml^−1^ TE), (*5*) (4 µg ml^−1^ of AgNPs + 0.004 µg ml^−1^ AMP or 0.125 µg ml^−1^ K or 0.008 µg ml^−1^ TE). *AMP* ampicillin, *K* kanamycin, *TE* tetracycline

**Fig. 9:**
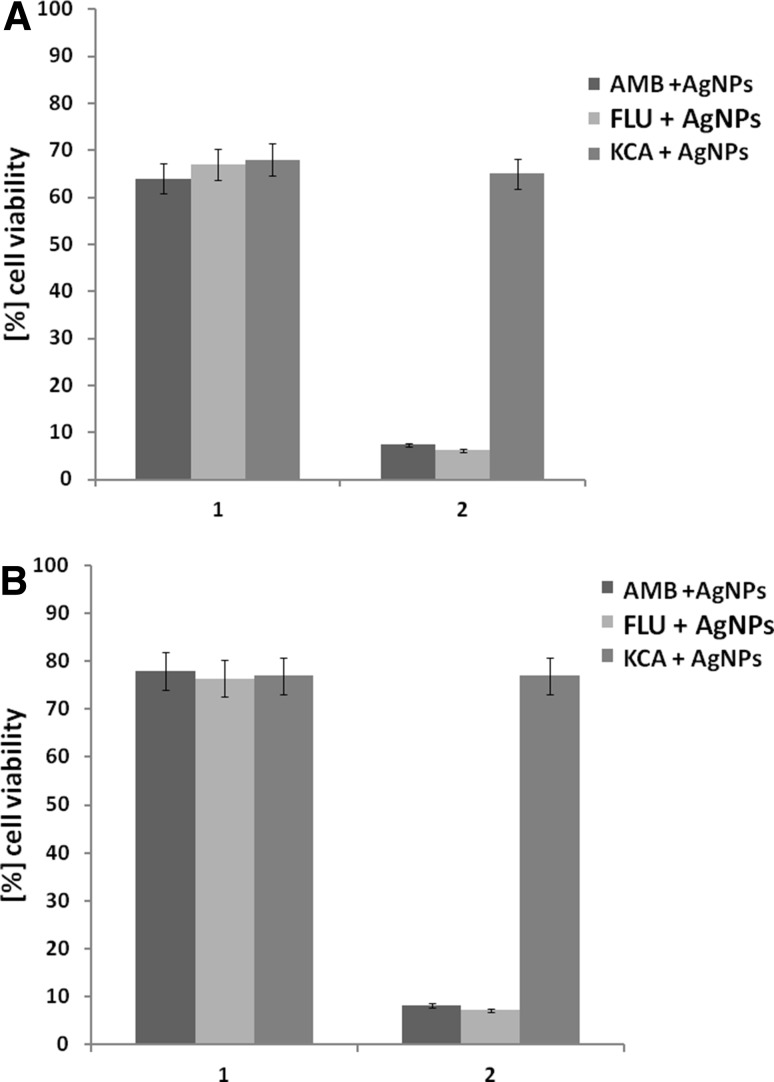
Cytotoxic activity of combined antimicrobial agents (AgNPs and antifungal antibiotics) against **a** mouse fibroblasts (3T3) and **b** HeLa cell line. Both antimicrobials were used at concentrations of their FIC (see Table 2): (*1*) (2 µg ml^−1^ of AgNPs + 0.016 µg ml^−1^ AMB or 64 µg ml^−1^ FLU or 64 µg ml^−1^ KCA), (*2*) (32 µg ml^−1^ of AgNPs + 1024 µg ml^−1^ AMB or 4 µg ml^−1^ FLU, and 2 µg ml^−1^ of AgNPs and 64 µg ml^−1^ KCA). *AMB* amphotericin B, *FLU* fluconazole, *KCA* ketoconazole

The cytotoxicity of AgNPs combined with selected antibacterial and antifungal antibiotics confirmed that four doubling reduction of both antimicrobial dosages caused reduction their cytotoxicity towards mammalian cells to value equal to IC_50_ of AgNPs used alone (Figs. 8, 9).

## Discussion

### Synthesis of AgNPs from *S. xinghaiensis* OF1 strain

In the present study, a colour change of reaction mixture resulted from excitation of surface plasmon resonance vibration of AgNPs present in the reaction mixture (Rai et al. 2015; Shanmugaiah et al. 2015) and was previously observed by many researchers (Kim et al. 2011; Golinska et al. 2015; Wypij et al. 2017). The UV–Visible spectroscopy is one of the most widely used techniques for structural characterization of nanoparticles (Annamalai and Nallamuthu 2016). The presence of absorbance peak in the range of 380–450 nm clearly indicates the formation of AgNPs in the solution (Ghorbani 2013).

### Physico-chemical characterization of AgNPs synthesized from *S. xinghaiensis* OF1 strain

Biosynthesized nanoparticles were similar to those reported by Wypij et al. (2017). Authors reported presence of small (4–24 nm), spherical and polydispersed AgNPs synthesised from actinobacterial strain. However, other studies on synthesis of AgNPs from actinobacteria revealed fabrication of bigger NPs of 55 nm (Rathod et al. 2016) or 44 nm (Składanowski et al. 2016), and from fungi (*Phoma gardeniae*) of 88 nm (Rai et al. 2015). Moreover, TEM characterization of biosynthesized AgNPs revealed that the edges of the nanoparticles were lighter than their centers, which indicates that nanoparticles were capped with biomolecules (e.g. proteins) (Annamalai and Nallamuthu 2016). Generally, it has been found that reducing the size of AgNPs enhances their stability and biocompatibility (Kim et al. 2011). From the above observations, it has been concluded that actinobacteria have potential for synthesis of small particles with large surface area.

The average size and concentration of biosynthesized AgNPs was also estimated by using nano tracking analysis (NTA). The NTA is based on light scattering and Brownian motion properties in order to measure the good sizing accuracy and comparatively narrow scatterings from monodispersed samples (Potara et al. 2015).

The difference in the size of biosynthesized AgNPs analysed by TEM (5–20 nm) and NTA (64–69 nm) resulted from fact that both techniques are based on different physical principles. During TEM analysis dried particles are measured and results present diameter of their metallic core only, whereas the NTA measure the hydrodynamic radius of nanoparticles in the solution and obtained nanoparticle size is always larger (Rai et al. 2015).

The zeta potential measurements, which revealed negative charge of biosynthesized AgNPs from OF1 strain clearly confirmed their higher stability than those reported by Prakasham et al. (2012) who observed zeta potential of AgNPs synthesized from *Streptomyces albidoflavus* as − 8.5 mV. The higher the negative or positive zeta potential, the stronger the prevention of AgNPs from its aggregation (Muthuvel et al. 2014).

FTIR measurements of biogenic AgNPs from OF1 strain provided evidence of specific functional groups constituting organic molecules, which are responsible for the reduction of silver ions to AgNPs and stability of AgNPs (Sanghi and Verma 2009; Manivasagan et al. 2013). The absorbance peaks at 3432 cm^−1^ can be assigned to N–H stretching vibration (Mohanta and Behera 2014; Shanmugaiah et al. 2015), whereas at 2925 cm^−1^ can be associated to C–H stretching vibration-asym (Karthik et al. 2013). The band at 1631 cm^−1^ can be attributed to the C=C stretching vibration (Annamalai and Nallamuthu 2016). The peak at 1385 cm^−1^ correspond to C–H sym. deformation vibration (Deepa et al. 2013) and at 1033 cm^−1^ can be assigned to C–O stretching vibration (Rai et al. 2015). Based on these results, it can be concluded that AgNPs were capped with organic compounds with amino bonds.

### Antimicrobial activity of AgNPs synthesized from *S. xinghaiensis* OF1 strain

Nanoparticles are considered to be a viable substitute to antibiotics and appear to have a high potential to solve the problem of the rise of microbial multidrug-resistance (Vandeputte et al. 2012; Franci et al. 2015). In the present study also AgNPs synthesized from *S. xinghaiensis* OF1 strain demonstrated antimicrobial activity against all tested bacteria and yeasts.

It is well known that antimicrobial activity of metal nanoparticle depends on their size and shape (Pal et al. 2007; Sharma et al. 2009) as well as stability (Shrivastava et al. 2007). Smaller nanoparticles cause higher toxicity to microbial pathogens, as these nanoparticles probably diffuse more easily than larger ones (Panáček et al. 2006; Mohan et al. 2007). It is claimed that for nanoparticles to be effective their size typically should be no larger than 50 nm (Dakal et al. 2016).

The AgNPs synthesized from *S. xinghaiensis* OF1 strain demonstrated more activity against Gram-negative bacteria and yeasts as compared to some selected antibiotics. Gurunathan and coworkers (2014) also reported higher antibacterial activity of AgNPs, which were synthesized from leaf extract of *Allophylus cobbe* against *Pseudomonas aeruginosa, Shigella flexneri, S. aureus* and *Streptococcus pneumoniae* than commercial antibiotics, namely ampicillin, chloramphenicol, erythromycin, gentamicin, tetracycline, and vancomycin. The MIC of AgNPs from OF1 strain against *E. coli, P. aeruginosa* and *B. subtilis* was found to be much lower than reported by Singh et al. (2013) who observed MIC of biogenic AgNPs against panel of Gram-negative bacteria (*E. coli, Acinetobacter baumannii, Enterobacter aerogenes, P.aeruginosa, Salmonella typhimurium* and *Shigella sonneii*) in the range of 150–600 μg ml^−1^, while against Gram-positive *S. aureus* and *Streptococcus mutans* > 1000 μg ml^−1^. Nasrollahi et al. (2011) reported higher antifungal activity of AgNPs against *C. albicans* than fluconazole and amphotericin B, but in our studies antifungal activity of amphotericin B was found in much lower concentration than fabricated AgNPs. Moazeni et al. (2012) reported antifungal activity of mycogenic AgNPs from *Trichophyton mentagrophytes* against *C. albicans*. The MIC value against tested fungi was found to be 4 μg ml^−1^, thus eightfold lower than in the present study (32 µl ml^−1^). However, Anasane et al. (2016) showed higher MIC of AgNPs synthesized from actinobacterial strain against *C. albicans* and *C. tropicalis* (40 μg ml^−1^, both). The MIC value of AgNPs against *M. furfur* (30 μg ml^−1^) was similar to findings in the present study.

In general, biocidal concentration of AgNPs against Gram-negative bacteria was much lower than all test antibiotics. Similar findings were recorded for fungi, with exception of amphotericin B.

The AgNP exposure to microorganisms causes irreversible changes in cell wall structure resulting in its disruption and affects integrity of lipid bilayer, permeability of the cell membrane and proper regulation of transport activity through the plasma (Panáček et al. 2006; Ghosh et al. 2012; Chauhan et al. 2013). AgNPs can further penetrate inside the microbial cell and interact with cellular structures and biomolecules such as proteins, lipids, and DNA. Interactions with cellular structures and biomolecules have damaging effect on microbes (Morones et al. 2005; Jung et al. 2008; Rai et al. 2012). Silver ions, as a secondary oxidation process of AgNPs, contribute to the biocidal properties of AgNPs by affecting transport and release of potassium (K^+^) ions from the microbial cells (Morones et al. 2005; Manivasagan et al. 2013). The increase in membrane permeability may lead to more pronounced effects such as loss by leakage of cellular contents, including ions, proteins, reducing sugars and sometimes cellular energy reservoir (ATP) (Lok et al. 2006; Kim et al. 2011; Li et al. 2013).

The synergistic activity of AgNPs with antimicrobials was observed against *B. subtilis, S. aureus E. coli* and *K. pneumoniae*. Contrary to antibacterial studies, the reports on determination of antimicrobial effect of AgNPs in combination with antibiotics by estimation of FIC index are scarce. Disc diffusion assay is most widely used method. In particular, data related to antifungal activity of AgNPs combined with antifungal agents are limited Synergistic activity of AgNPs and antibiotics (e.g. ampicillin, chloramphenicol, gentamycin, kanamycin, neomycin) against *S. aureus*, including MRSA, *Streptococcus mutans, E. coli, P. aeruginosa* etc. have been reported (Prakasham et al. 2012; Muthuvel et al. 2014; Potara et al. 2015). Authors demonstrated various effects of AgNP and antibiotic combinations from synergistic to antagonistic. In the present study, the synergistic and non-synergistic effect of combination of biosynthesized AgNPs with antibiotics was recorded. Interestingly, the synergistic effect was also observed for ampicillin or kanamycin and AgNPs against *E. coli* or ampicillin and AgNPs against *K. pneumoniae*, even though MICs of those antibiotics were not determined for bacteria (> 1024 µg ml^−1^). The use of the highest tested concentration of antibiotic resulted in high synergistic effect of combined antimicrobials against test strains. Similar effect was also noted for fluconazole and ketoconazole in presence of AgNPs against *C. albicans* or ketoconazole in combination with AgNPs against *M. furfur*.

Unlike above results, *P. aeruginosa* showed resistance to ampicillin and *M. furfur* to amphotericin B, and combination of antibiotic with AgNPs did not affect susceptibility of microbial cells to tested antimicrobial agents. The activity of combined antimicrobials was determined as indifferent and non-synergistic effect, respectively.

It is claimed that organic compounds present on the surface of biogenic AgNPs interact with the antibiotics, modifying the output properties. The enhanced antimicrobial effect of combination of AgNPs with antibiotics may be caused by a binding between an antibiotic and biogenic nanosilver. The tested antibiotic contain many hydroxyl and amino groups that can easily react with AgNPs by chelation. It is also postulated that AgNPs act as an antibiotic carrier, facilitating penetration into microbial cells and improve their antimicrobial action (Duran et al. 2011). Moreover, combination of AgNPs with antibiotic reduces the toxicity of both compounds toward human cells by decreasing the required dosage with enhanced antimicrobial properties, which was also observed in the present study. Authors also claimed that such combination restores the ability of the drug to kill bacteria that have acquired resistance to them (Allahverdiyev et al. 2011; Singh et al. 2013, 2015). Combined use of AgNPs and antibiotic/antifungal agent in the present studies supported above findings and proved that such combinations allow to reduce dosage of both antimicrobials and their toxicity toward mouse fibroblasts and HeLa cells.

### Cytotoxicity of AgNPs synthesized from *S. xinghaiensis* OF1 strain

The in vitro study of cytotoxic efficacy of AgNPs synthesized from *S. xinghaiensis* OF1 strain on mouse fibroblasts (3T3) and HeLa cell line was high (IC_50_ about 4 µg ml^−1^), which was also confirmed by biocompatibility index determination. However, other authors reported that concentration of AgNPs from actinobacterial strains required for reduction in viability by 50% HeLa cells was 200 or 100 µg ml^−1^ (Manivasagan et al. 2013; Rathod et al. 2016). The lower cytotoxic effect of actinobacterial synthesized AgNPs (IC_50_ value of 64.5 µg ml^−1^) against mouse fibroblasts (L929 cell line) was found in studies by Składanowski et al. (2016). HeLa cell lines are commonly used model, but it is well known that the cancer cells show lower sensitivity to cytotoxic conditions than the more physiologically relevant 3T3 cell line. In our opinion, the inconsistencies between other researchers and data of the present study may arose due to the differences in experimental protocols and models (Julien et al. 2010; Nogueira et al. 2011).

Panáček et al. (2016) showed that in some cases combination of antibiotics with AgNPs at their concentrations equal to MIC values decreased cell viability from 85 to 71% when compared to untreated cells. Authors concluded that the cytotoxic effect was higher as a result of the additive cytotoxic effects of the antibiotics and AgNPs. The highest cytotoxic effect was detected for ampicillin/sulbactam, cefazolin, meropenem and chloramphenicol combined with AgNPs. We report that low concentrations of antibiotics and AgNPs slightly decreased the viability of cells (to 90–95%) in comparison with the control cells depending on the antibiotics used.

On the basis of the results, we suggest that biogenic AgNPs have much potential for application as an antimicrobial agent in combination with antibiotics/antifungals. Combined therapy could significantly decrease concentration of both antimicrobials and remove the resistance of antibiotics to pathogenic microbes.
